# Pattern and presentation of cardiac diseases among patients with chronic kidney disease attending a national referral hospital in Uganda: a cross sectional study

**DOI:** 10.1186/s12882-015-0128-z

**Published:** 2015-08-04

**Authors:** Christopher Babua, Robert Kalyesubula, Emmy Okello, Barbara Kakande, Erias Sebatta, Michael Mungoma, Charles Mondo

**Affiliations:** Department of Medicine, Faculty of Medicine, Gulu University, Gulu, Uganda; Nephrology Division, Department of Medicine, Makerere University, College of Health Sciences, Kampala, Uganda; Uganda Heart Institute, Kampala, Uganda; Cardiology Division, Department of Medicine, Mulago National Referral Hospital, Kampala, Uganda

## Abstract

**Background:**

Chronic kidney disease is a risk factor for development of cardiovascular diseases. Cardiovascular diseases are the primary cause of morbidity and mortality in patients with chronic kidney disease. There is limited data on cardiovascular diseases among chronic kidney disease patients in resource limited settings including Uganda. We determined the prevalence and patterns of cardiac diseases among patients with chronic kidney disease attending the nephrology outpatient clinic in Mulago National Referral Hospital in Uganda.

**Methods:**

This was a cross sectional study in which two hundred seventeen patients with chronic kidney disease were recruited over a period of 9 months. Data on demographic characteristics and risk factors for cardiovascular diseases were collected using a standardized questionnaire. Cardiac evaluation was done using resting electrocardiography and transthoracic echocardiography performed for all study participants and findings entered into a data sheet.

**Results:**

One hundred eleven (51.2 %) of the 217 participants were male. Mean age was 42.8 years. One hundred eighteen (54.4 %) of patients had either eccentric or concentric left ventricular hypertrophy. Patients with left ventricular hypertrophy were more likely to be hypertensive (*p* <0.001) or anemic (*p* = 0.034). Up to 9.2 % of study subjects had valvular heart disease (rheumatic or degenerative) and 22 % had pericarditis. Forty one patients (18.9 %) had left ventricular systolic failure (Ejection fraction < 50 %). There was a higher prevalence of systolic failure in patients with left ventricular hypertrophy (21 % *vs.* 16 %) although this was not statistically significant, *p* = 0.346. Thirty eight participants (17.5 %) had diastolic failure while 2 % had cardiac rhythm abnormalities.

**Conclusion:**

Cardiac abnormalities are common in a predominantly young African population with CKD. Clinicians should routinely screen and manage cardiovascular disease in CKD patients.

## Background

Cardiovascular disease is the primary cause of morbidity and premature mortality in chronic kidney disease [1, 2]. The high risk of cardiovascular morbidity and mortality in end stage renal disease (ESRD) is a well established fact [3]. However a high rate of both fatal and non fatal cardiovascular events has been observed in patients with earlier stages of chronic kidney disease [4]. Cardiac diseases have not been extensively studied among patients with CKD in sub-Saharan Africa.

Data drawn from more than 20,000 subjects enrolled in different studies (Cardiovascular Health Study, Framingham Heart and others) showed association between CKD and a number of adverse cardiovascular outcomes. CKD was found to be an independent risk factor for MI, stroke, and death after exclusion of patients with base line cardiovascular disease. Furthermore, blacks were noted to be at a higher risk than Caucasians [5].

The development of CKD is a risk factor for adverse cardiovascular events in patients with hypertension and normal base line renal function. This was demonstrated in a prospective cohort study involving 281 subjects with essential hypertension and normal base line kidney function in whom adverse cardiovascular events including acute myocardial infarction (AMI), heart failure, stroke, and/or death were more likely to occur in those who developed CKD (15 %) in the 13 years of follow up (41 % adverse events in CKD *versus* 13 % in normal kidney function, HR 2.5, CI 95 % 1.3-4.8) [6].

A study done in the University of Nigeria teaching hospital found LVH prevalence of 95.5 % among CKD patient scompared with 6.7 % in controls [7]. Hypertension was present in 85.2 % of participants. The prevalence and severity of LVH increases with decreasing GFR/Renal function [8, 9] occurring in 30–45 % of patients with CKD not on dialysis in the USA.

Hypertension, LVH and heart failure are some of the causes of arrhythmias in CKD. However a Japanese study that enrolled 1118 patients with hypertension showed that the development of CKD was a powerful predictor for new onset atrial fibrillation (*p* = 0.009) independent of LVH and left atrial enlargement [10].

Uremia as well as other factors prevalent in CKD including hypertension, hyperlipidemia, DM, malnutrition, inflammation, hypertrophic cardiomyopathy, anaemia among others [11] result in extaosseous calcifications that may involve the cardiac valves. These calcifications result in valvular stenosis and/or regurgitation.

Pericardial disease including pericarditis and pericardial effusion are relatively common in patients with renal failure. Uremic pericarditis is observed in 6–10 % of patients with advanced renal failure [12].

In sub-Saharan Africa, there is limited data on the occurrence of cardiovascular diseases among patients with CKD. The available studies on CKD patients show a younger population as compared with the western studies and this age difference may result in a difference in cardiovascular disease epidemiology between the two study settings. Furthermore the limited access to renal replacement therapy in sub-Saharan Africa may impact on the relative prevalence of cardiac dysfunction among patients with CKD in the two study settings.

## Methods

We conducted a cross sectional study between June 2012 and February 2013 at Mulago National Referral Hospital in Kampala, Uganda. The hospital is located in central Uganda, East Africa and doubles as the teaching hospital for Makerere University’s college of health sciences. It serves the 33million people of Uganda as well as Referrals from the neighboring Eastern Democratic Republic of Congo and the Republic of South Sudan.

We consecutively recruited adults with CKD aged 18 years and above. An online sample size calculator by survey systems (http://www.surveysystem.com/sscalc.htm) was used to determine sample size. Considering a nephrology outpatient clinic population of 500 patients, we calculated a total sample size of 217 subjects to enable estimates to a precision of 5 %.

All patients attending the nephrology outpatient clinic were invited to participate in the study as potential respondents. Only patients with evidence of kidney disease (serum creatinine >1.4 mg/dL for males and >1.2 mg/dL for females) for ≥3 months as well as those with impaired renal function in the setting of atrophic kidneys (<8 cm in length), were recruited. Patients who had any form of renal replacement therapy (Hemodialysis, peritoneal dialysis or renal transplant) were excluded from the study.

Ethical approval was obtained from the School of Medicine Research and Ethics Committee of the College of Health Sciences, Makerere University. A written informed consent was obtained from all study participants before recruitment into the study.

A standardized pretested questionnaire was used to collect data on socio-demographic characteristics, medical history, and physical signs with emphasis on cardiovascular risk factors, laboratory test parameters, electrocardiography (ECG) and echocardiography variables. ECG was done primarily to evaluate cardiac rhythm with particular interest in atrial fibrillation (absent P waves). ECG changes suggestive of ischemic heart disease (Q waves, S-T and T wave changes) were also evaluated. Echocardiography was performed to evaluate cardiac structure and function. Left ventricular hypertrophy (Interventricular septum and/or left ventricular posterior wall diameter >11 mm), ischemic heart disease (regional wall motion abnormalities), valvular heart disease, pericarditis (pericardial thickening and/or effusion), left ventricular systolic failure (Left ventricular ejection fraction <50 %), left ventricular diastolic failure (E/A ratio <1), and pulmonary artery hypertension (PAH) (Tricuspid regurgitation pressure gradient/TRPG >35 mmHg) were the key parameters studied at echocardiography.

Rest ECGs were done using the Schillar ECG Recorder, (Basal Switzerland). Echocardiograms were done using Vivid 7 Dimension, GE Medical Systems (Horten, Norway).

### Statistical analyses

Data was double entered into epidata version 3.1 and exported to STATA version 10 (after validation) for analysis. Results were expressed as percentages (frequencies of the different cardiac diseases, stratification of respondents by stage of CKD) and means (age) with standard deviations and presented in tables and graphs. *Chi*^*2*^ tests were used to determine associations (LVH and anemia, LVH and hypertension, cardiac failure and LVH, determination of trends *i.e.,* variation of frequencies of each variable across the different CKD stages). Results were statistically significant when the *P* value was <0.05.

## Results

**Fig. 1:**
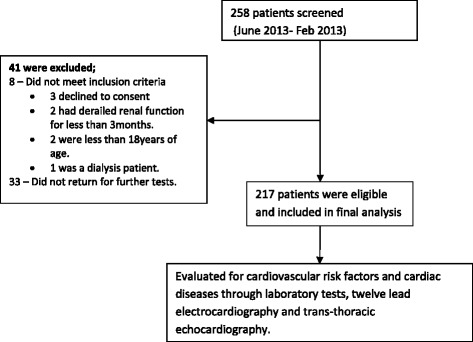
Patient/participant flow chart showing number of patients screened, number excluded (with reasons for exclusion) and number recruited into the study

**Fig. 2:**
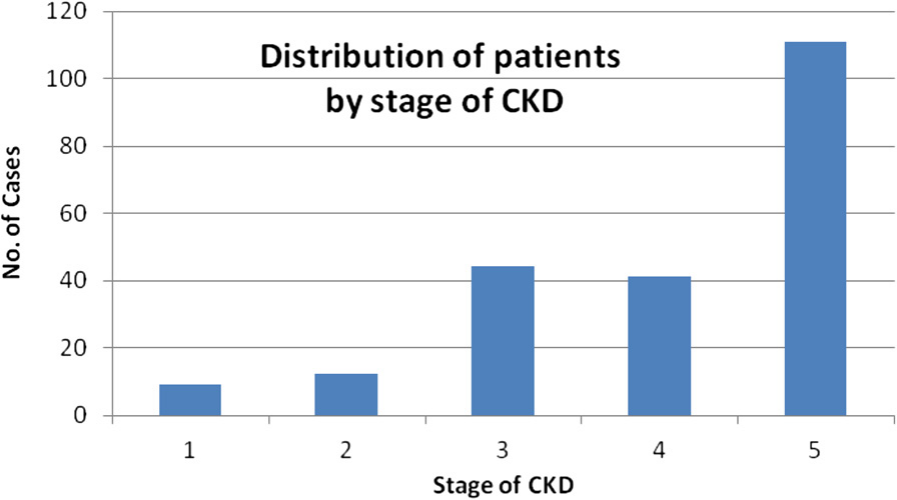
Graph showing distribution of patients by stage of CKD. *GFR* glomerular filtration rate

**Fig. 3:**
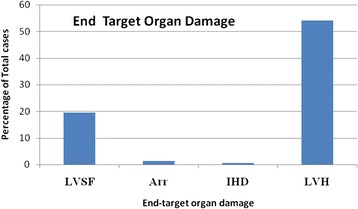
Graph showing the proportion of the different cardiac pathologies among the study participants. *LVSF* left ventricular systolic failure, *Arr* arrhythmia, *IHD* ischemmic heart disease, *LVH* left ventricular hypertrophy

A total of 258 patients were screened over a period of nine months. Forty one were excluded from the study for various reasons (Fig. 1). One hundred and six participants (106, 49 %) enrolled in the study were females. The mean age of study participants was 42.8 years (95 % CI = 40.6–44.9). About half of the patients had end stage renal disease (111, 51.2 %) but were not on renal replacement therapy (Fig. 2). A total of 184 patients (84.8 %) had proteinuria. One hundred sixty two patients (162, 74.65 %) had a non-reactive HIV antibody test within the past three months of recruitment, thirty two patients (32, 14.75 %) were HIV positive, while the remaining twenty three (23, 10.60 %) had no evidence for the HIV test in the past three months. The patient characteristics are summarized in Table 1. Twenty five patients (11.5 % of participants) had a positive history of cigarette smoking. Twenty two (10.1 %) were obese while 35 (16.2 %) had diabetes mellitus. Eighty nine participants (41 %) had high non-HDL cholesterol (≥130 mg/dL). One hundred fifty six participants (71.9 %) were anemic with hemoglobin concentration <11 g/dL (Fig 3).

**Table 1:** Demographic and clinical characteristics of study participants

Characteristic	Frequency (*N* = 217)	Percentage (%)
Age		
<45 years	124	57.1
45-59	59	27.2
≥60	34	15.7
Sex		
Female	106	48.9
Stage of CKD		
1 (GFR ≥90 mL/min/m^2^)	9	4.2
2 (GFR 60–89 mL/min/m^2^)	12	5.5
3 (GFR 30–59 mL/min/m^2^)	44	20.3
4 (GFR 15–29 mL/min/m^2^)	41	18.9
5 (GFR <15 mL/min/m^2^)	111	51.2
Proteinuria		
Present	184	84.8
HIV antibody test status		
Non-reactive	162	74.7
Reactive	32	14.8
Not available	23	10.6

*GFR* glomerular filtration rate, *HIV* Human Immunodeficiency Virus

### Prevalence and patterns of cardiac diseases

In this study, 118 patients (54.4 %) were found to have either concentric or eccentric LVH defined as left ventricular posterior wall and/or interventricular septal thickness greater than 11 mm measured from leading to trailing edge. Patients with LVH were more likely to be hypertensive (114, 96.6 % *vs.* 4, 5.4 % of 191), p < 0.001. Furthermore patients with LVH were more likely to be anemic (92, 77.8 % with Hb < 11 g/dL *vs.* 26, 22.0 % with Hb ≥11 g/dL) *p* = 0.034. Only one patient (1, 0.5 %) was found to have a hypokinetic left ventricular posterior wall with moderate left ventricular systolic failure (left ventricular ejection fraction of 34 %) described as ischemic cardiomyopathy. The patient had no history of treatment for angina or any of the coronary syndromes. Twenty patients (20, 9.2 %) were found to have valvular heart disease. Three of the twenty patients had valvular abnormalities consistent with rheumatic heart disease (RHD). The other seventeen patients had degenerative/atherosclerotic changes of cardiac valves involving mostly the aortic or mitral valve. Forty seven patients (47, 21.7 %) had echocardiographic evidence of pericarditis suggested by pericardial effusion, pericardial thickening or both. Patients with pericarditis were more likely to have a serum urea concentration greater than 60 mg/dL, *p* = 0.327. (85 % with pericarditis had urea > 60 mg/dL *vs.* 71 % with normal pericardium and urea > 60 mg/dL). None of the patients with pericarditis had evidence of cardiac tamponade. Forty one patients (41, 18.9 %) were found to be in left ventricular systolic failure ranging from mild to severe failure (*i.e.,* left ventricular ejection traction <50 %). There was a higher prevalence of systolic failure among patients with LVH (25 % *vs.* 16 %) although this was not statistically significant, *p* = 0.346. A small proportion of patients, thirty eight (38, 17.5 %) had left ventricular diastolic dysfunction (LVDD). Patients with LVDD were more likely to have LVH although this was not statistically significant. Four patients (4, 1.8 %) were found to have cardiac rhythm disturbances with two having atrial fibrillation (Tables 2 and 3).

**Table 2:** Cardiac diseases among patients attending the Mulago renal outpatient clinic

Variable	Frequency (*N* = 217)	Percentage
LVH	118	54.4
PAH	48	22.1
Pericarditis	47	21.7
LV systolic failure	41	18.9
LV diastolic failure	38	17.5
Valvular heart disease	20	9.2
Cardiac dysrrhythmias	4	1.8
Ischemic heart disease	1	0.5

*LVH* left ventricular hypertrophy, *LV* left ventricle, *PAH* pulmonary artery hypertension

**Table 3:** Variation of frequency of cardiac diseases across the different CKD stages

Variable	CKD stage					*p* value
	I (*n* = 9)	II(*n* = 12)	III(*n* = 44)	IV(*n* = 41)	V(*n* = 111)	
LVH *n* (%)	0(0.00)	4(33.3)	19(43.2)	24(58.5)	71(64)	0.001
IHD *n* (%)	0(0.00)	0(0.00)	1(2.3)	0(0.00)	0(0.00)	0.423
VHD *n* (%)	0(0.00)	0(0.00)	6(13.6)	1(2.4)	13(11.7)	0.182
Pericarditis *n* (%)	2(22.2)	0(0.00)	5(11.4)	11(26.8)	29(26.9)	0.091
LVSF *n* (%)	0(0.00)	1(8.3)	8(18.2)	8(19.5)	24(21.6)	0.473
LVDD *n* (%)	0(0.00)	3(25)	13(29.6)	6(14.6)	16(14.4)	0.101
Arrhythmias *n* (%)	1(11.1)	0(0.0)	1(2.3)	1(2.4)	1(0.9)	0.270

*LVH* left ventricular hypertrophy, *IHD* ischemic heart disease, *VHD* valvular heart disease, *LVSF* left ventricular systolic failure, *LVDD* left ventricular diastolic dysfunction

## Discussion

One hundred eighteen patients accounting for 54.4 % of study participants were found to have either eccentric or concentric left ventricular hypertrophy. This proportion is significantly lower than that found in other studies including one in Nigeria where 95.5 % of patients with CKD were found to have LVH at first nephrology consult [7]. Similarly there was a higher prevalence of LVH among Albanian CKD patients at 81.9 % [13]. The reasons for this remarkable difference are not clear. Differences in determination of LVH where left ventricular wall thickness was used in this study as compared to left ventricular mass index in the Nigerian study may partly account for these differences. This difference cannot be explained by age since the mean age of patients in the Nigerian study was 42 years, similar to the 42.8 years in this study. Differences in treatment of hypertension including use of ACE inhibitors which could probably account for this variation need to be evaluated in further studies.

Anemia and LVH are known causes of systolic failure or dysfunction. Both occur commonly among patients with CKD including those in this study. However the association of LVH and anemia with systolic failure was not statistically significant in this study most likely due to the relatively small sample size. Arodiwe *et al.* reported left ventricular systolic dysfunction in 15.1 % of CKD patients [14]. This finding is similar to the one in the current study. This similarity is probably due to similarity in patient populations. There was a lower prevalence (17.5 %) of LVDD in this study as compared with one done by Arodiwe *et al.* in Nigeria where the prevalence was at 62.8 % in CKD patients [15]. The reasons for this remarkable difference are unclear. Differences in prevalence of LVH are possible explanations.

Despite the relatively small proportion of patients with evidence of ischemic heart disease, the development of CKD is a known risk factor for acute myocardial infarction (AMI) [6]. Data from sub Saharan Africa about IHD in CKD are lacking.

Pericarditis presumed to be of uremic etiology was quite common among the study participants affecting up to 21.7 %. Rostand *et al.* demonstrated a smaller proportion of pericarditis among patients with End Stage Renal Disease (ESRD) at 6–10 % [12]. The relatively higher prevalence of pericarditis in this study could be explained by the high proportion of ESRD patients who are not on any renal replacement therapy.

The most studied rhythm disturbance in CKD is atrial fibrillation. In this study, only two patients (0.9 %) were found to have atrial fibrillation. A study done in the USA found a much higher atrial fibrillation prevalence of 21.2 % in nondialysis CKD patients [16]. This difference is probably due to age difference between the study populations. In the American study, the mean age of patients with atrial fibrillation was higher than that for patients with sinus rhythm (76 ± 11 *versus* 63 ± 15 years). These mean ages are both higher than the 42.8 year mean age for the subjects in this study.

It is a well established fact that worsening renal failure is associated with an increasing frequency of both cardiovascular risk factors and cardiac diseases. However the patient population in this study was not adequate for observation of trends across the different stages of CKD.

In this study, renal biopsies were not done and thus the exact nature of kidney damage/pathology was not ascertained and analysis therefore done for CKD as a block as opposed to clusters by etiology or nature of underlying pathology.

## Conclusion

This study has demonstrated the common occurrence of abnormalities of cardiac structure and function among patients with CKD. The patients in this study are generally younger than the CKD patients seen in the western studies. The large number of patients with ESRD not on renal replacement therapy could partly account for the high burden of cardiac diseases in this relatively young patient population.

## Abbreviations

AF: Atrial Fibrillation
AMI: Acute Myocardial Infarction
CAD: Coronary Artery Disease
CBC: Complete Blood Count
CHF: Congestive Heart Failure
CKD: Chronic Kidney Disease
CRP: C - reactive protein
CVD: Cardiovascular Disease
DM: Diabetes Mellitus
ECG: Electrocardiography
HHD: Hypertensive Heart Disease
GFR: Glomerular Filtration Rate
IHD: Ischemic Heart Disease
IVS: Inter Ventricular Septum
LVH: Left Ventricular Hypertrophy
NKF-K/DOQI: National Kidney Foundation-Kidney Disease Outcomes Quality Initiative
RFT: Renal Function Tests
